# Larval Transport Modeling of Deep-Sea Invertebrates Can Aid the Search for Undiscovered Populations

**DOI:** 10.1371/journal.pone.0023063

**Published:** 2011-08-08

**Authors:** Jon M. Yearsley, Julia D. Sigwart

**Affiliations:** 1 School of Biology and Environmental Science, University College Dublin, Belfield, Dublin, Ireland, United Kingdom; 2 School of Biological Sciences, Queen's University Belfast, Marine Laboratory, Portaferry, Northern Ireland, United Kingdom; California Academy of Sciences, United States of America

## Abstract

**Background:**

Many deep-sea benthic animals occur in patchy distributions separated by thousands of kilometres, yet because deep-sea habitats are remote, little is known about their larval dispersal. Our novel method simulates dispersal by combining data from the Argo array of autonomous oceanographic probes, deep-sea ecological surveys, and comparative invertebrate physiology. The predicted particle tracks allow quantitative, testable predictions about the dispersal of benthic invertebrate larvae in the south-west Pacific.

**Principal Findings:**

In a test case presented here, using non-feeding, non-swimming (lecithotrophic trochophore) larvae of polyplacophoran molluscs (chitons), we show that the likely dispersal pathways in a single generation are significantly shorter than the distances between the three known population centres in our study region. The large-scale density of chiton populations throughout our study region is potentially much greater than present survey data suggest, with intermediate ‘stepping stone’ populations yet to be discovered.

**Conclusions/Significance:**

We present a new method that is broadly applicable to studies of the dispersal of deep-sea organisms. This test case demonstrates the power and potential applications of our new method, in generating quantitative, testable hypotheses at multiple levels to solve the mismatch between observed and expected distributions: probabilistic predictions of locations of intermediate populations, potential alternative dispersal mechanisms, and expected population genetic structure. The global Argo data have never previously been used to address benthic biology, and our method can be applied to any non-swimming larvae of the deep-sea, giving information upon dispersal corridors and population densities in habitats that remain intrinsically difficult to assess.

## Introduction

The habitat connectivity, spatial diversity, and distribution of deep-sea benthic animals are largely determined by the dispersal capacity of their pelagic larvae [1]. Yet a major challenge in studying these marine animals is the availability of data, because the deep benthic environment is remote and largely unexplored in comparison to its total scale [2], [3]. A growing number of studies have documented the reproductive ecology of deep marine organisms through field collection (e.g. [4]) and laboratory culture (e.g. [5]). Such modern work has demonstrated the diversity of life history strategies in the deep sea, which are as varied as their shallow counterparts ([6] contra [7]). However, free-floating larvae in deep-sea waters remain very challenging to study, and the new method presented here represents a step forward in assessing the motility of unobserved deep-sea larvae via inference from computational modelling.

Faced with sparse data on deep-sea dispersal ecology, modelling approaches, such as larval transport models [8], offer the possibility of combining diverse data sets to make novel, testable predictions. The majority of quantitative work on larval transport models to date has focussed on shallow-water organisms and coastal currents [9], [10]. There are two substantial factors that differentiate deep-sea dispersal from shallow water models: basin scale current dynamics and local temperature. In the colder water of the deep sea, non-feeding larvae may have a significantly extended lifespan due to metabolic reduction [11], [12]. However, despite an assumed ‘paradigm’ of open deep-sea dispersal that could allow larval transport over large distances in ocean basins, slower moving deep ocean currents may present a barrier to larval dispersal [7]. Work on deep-sea connectivity has mainly focussed on hydrothermal vent fauna (e.g. [5], [13], [14]), but other habitats, such as sunken wood, are also important habitat substrates for deep-sea fauna. Sunken wood is particularly important in the tropical Pacific [15], but it is also difficult to locate, variable in composition and remains under-studied [16].

In order to construct a realistic dispersal model, polyplacophoran molluscs (chitons) were used as the model organisms in our test case. Our dataset presents several particular advantages: organisms in this case endemic to an ephemeral but widely distributed habitat (sunken wood); weakly swimming larvae suitable for simple particle modelling; larvae that do not feed and therefore have a limited potential larval lifespan; known populations of morphologically conspecific adults across a broad range of south-west Pacific islands; and shallow water counterparts that allow for robust determination of life history variables. The study fauna is found on sunken wood distributed in depth from approximately 200 m to 1600 m across the tropical South Pacific [17], [18], [19], [20]. It is well established from shallow-water taxa that all known polyplacophorans have lecithotrophic trochophore larvae [21], [22]. Limited larval mobility of trochophores means that the individual larvae function effectively like drifting particles. Lecithotrophic (non-feeding) larvae are typically associated with shorter planktonic larval duration (PLD) than feeding larvae [12], [23]. Anecdotal evidence suggests that lecithotrophic trochophore larvae of certain taxa can persist for over 12 months [24], particularly in the depressed metabolic state of cold temperature environment. Yet open dispersal and passive, yet metabolically depressed and long-lived, larvae may not be sufficient to explain the connectivity already known from widely dispersed fauna common to fragmented deep-sea habitats. Our study aims to test whether this temperature-based proposition can reasonably be extended to include deep-sea habitats as well as colder coastal environments, or whether the deep sea is not in fact equal to the shore.

We present a new method and a new approach to the black box of deep-sea larvae, by applying a biological-physical model to study the dispersal of planktonic larvae from deep-sea benthic invertebrates. Our approach incorporates known species distribution data, predictive values of PLD for a model organism, and observational oceanographic current data from autonomous probes. This model empirically addresses the maximum potential dispersal distance among a widely distributed fauna, estimating realistic physical connectivity between known populations and the potential locations of undiscovered populations.

## Results

Our dispersal model indicated that chiton larvae generally cannot reach between the currently known adult populations on separate South Pacific archipelagos in a single generation. The model was built from consideration of several underlying variables, described below, that corroborated this general finding.

### Observed (starting) distribution of deep-sea chitons

Deep-sea chitons have been well reported from three major archipelagos in the south-west Pacific [17], [19]. We combined records from the work of two previous studies (Table 1; the bounding boxes of these locations are shown in Supporting Information S1). All of the taxa examined are in the order Lepidopleurida, which is represented in shallow water most commonly by the genus *Leptochiton*. All of the study species in our test case are known only from sunken wood or plant material [17], [19]. The distances between locations (Table 2), showed an order of magnitude difference in separation distance between locations within the same archipelago and locations from different archipelagos. We used the locations of these adult populations as sources of larval particles in our model.

**Table 1:** Study species of chitons identified from the seven collecting expeditions.

	Number of stations			
	NC	V	SI	Depth range (m)
*Ferreiraella plana* (Nierstrasz, 1905)	1	8	1	560–1040
*Ferreiraella xylophaga karenae* Sirenko, 2001	0	3	2	475–798
*Leptochiton boucheti* Sirenko, 2001	0	6	1	504–900
*Leptochiton deforgesi* Sirenko, 2001	0	2	7	520–977
*Leptochiton habei* Saito, 1997	0	1	3	395–780
*Leptochiton juvenis* (Leloup, 1981)	0	8	0	488–800
*Leptochiton* n. sp. 1 *sensu* Sigwart, 2008	0	0	3	977–1218
*Leptochiton* n. sp. 2 *sensu* Sigwart, 2008	0	4	0	630–705
*Leptochiton* n. sp. 3 *sensu* Sigwart, 2008	0	2	1	800–854
*Leptochiton* n. sp. 4 *sensu* Sigwart, 2008	0	1	4	358–623
*Leptochiton* n. sp. 5 *sensu* Sigwart, 2008	0	8	0	492–777
*Leptochiton vaubani* Kaas 1991	0	3	12	236–1118
*Leptochiton saitoi* Sirenko, 2001	15	1	2	210–1118
*Leptochiton thandari* Sirenko, 2001	0	5	7	236–1060
*Leptochiton vanbellei* Sirenko, 2001	1	11	5	454–1620
*Leptochiton vietnamensis* Sirenko 1998	2	1	4	316–1218
*Nierstraszella andamanica* (Smith, 1906)	0	4	25	200–1060
*Nierstraszella lineata* (Nierstrasz, 1905)	0	23	25	200–1060
Total number of species	4	17	15	200–1620

The distributional data from these expeditions was used to initialise our method. NC, New Caledonia (expeditions Bathus 1, Bathus 2, Bathus 4); V, Vanuatu (Boa 1, Musorstom 8); and SI, the Solomon Islands (Salomon 1, Salomon 2). Some species have cellulose-digesting bacteria present in the gut (M. Zbinden, pers. comm.) suggesting dependent endemism.

**Table 2:** Median dispersal distances and total path lengths (in kilometres) of simulated larvae.

	Solomon Islands	Vanuatu	New Caledonia
Distance from source (km)			
50 days	162	29	159
	(28–357)	(4–117)	(10–314)
100 days	285	40	378
	(46–539)	(4–186)	(13–552)
250 days	48	63	565
	(65–954)	(4–496)	(16–942)
500 days	560	74	692
	(69–1159)	(4–812)	(17–1554)
Path length (km)			
50 days	263	95	185
	(70–482)	(40 – 190)	(59–326)
100 days	545	185	441
	(154–910)	(75–363)	(120–668)
250 days	1315	456	1094
	(420–2131)	(176–893)	(314–1408)
500 days	2190	899	1834
	(844–4032)	(337–1686)	(635–2619)
Separation between island groups (km)			
Solomon Islands	180		
	(0.37–590)		
Vanuatu	1400	120	
	(930–1900)	(0.18–500)	
New Caledonia	1700	650	120
	(1200–2000)	(390–820)	(1.2–360)

Simulated larvae originated in the three archipelagos and travelling with ocean currents between 800 m and 1400 m deep. The 95% quantiles are shown in brackets. Initial larval distribution corresponds to the known distribution from field samples, with simulations originating from all sample locations (comparing dispersal and path lengths). Bottom, median distances in kilometres between the known populations of deep-sea molluscs within and between three island groups: in the Solomon Islands, Vanuatu and New Caledonia (minimum and maximum distances shown in brackets).

### Estimating deep-sea oceanographic currents

We used nine years of data (2001–2009) from the global Argo array [25] to estimate Eulerian velocity fields of the deep-sea ocean currents for two depth ranges (800–1400 m and 1400–2500 m) within the rectangular region 143°W–176°W, 1.5°S–25°S. For each depth range we constructed 500 velocity fields by randomly thinning the data before using the optimal interpolation method of Molcard [26]. We consider dispersal driven by the more shallow data partition, 800–1400 m to be more biologically realistic for our chiton model organism as it corresponds most closely with the majority of starting points. The construction of these vector fields is described in detail in the methods, below.

### Estimating planktonic larval duration

The final element underlying our model was the simulation time span, which needed to exceed the longest time that a chiton larvae could reasonable survive adrift in the water. We used the approach of O'Connor *et al.* [12] to estimate the planktonic larval duration (PLD) of chiton larvae from the water temperature. The general idea was to use data from lecithotrophic species found in shallow waters to fit a mixed-model of PLD against water temperature, and then to extrapolate to the water temperatures of the tropical Pacific where the deep-sea chitons were recorded.

**Figure 1:**
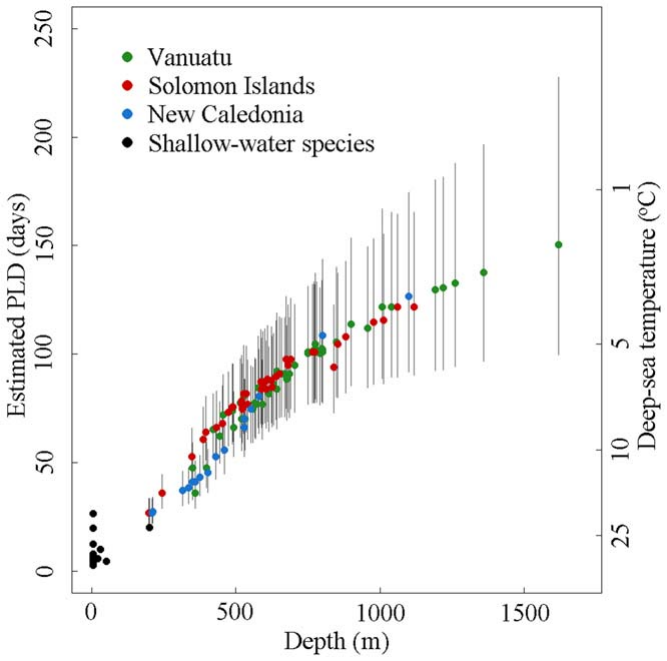
Observed and estimated planktonic larval durations (PLD) for shallow and deep-sea chiton species. Temperature-based estimates of PLD (in days) for the sampled adult habitats in the Solomon Islands (red), Vanuatu (green), and New Caledonia (blue), and observed chiton PLDs for 14 shallow water species (black). Deep-sea temperatures are taken from USGODAE Navy GDEM Monthly temperature recordings at the coordinates and depths of each collecting station (http://www.usgodae.org/las/getUI.do). PLD values are calculated from the temperature (T) using the population-averaged equation: ln(PLD) = 3.54−1.30·ln(T/15)−0.26·(ln(T/15))^2^ [12].

Our predictions of PLD (with 95% confidence intervals) for chiton larvae at the sampling locations ranged from 27 days (22–33 days) at a depth of 197 m to 151 days (100–225 days) at a depth of 1620 m (Fig. 1). Allowing for the possibility of an additional 50 days drift of the egg prior to larval hatching made our results at 250 days an upper limit for dispersal of a single larval cohort, and our results at 500 days relevant to dispersal across multiple generations.

### Simulating the dispersal pathways

For each of the three archipelagos where chitons have been recorded (the Solomon Islands, Vanuatu and New Caledonia) we used the estimated deep-sea ocean currents to create 10000 simulated paths of passively dispersing larvae over 500 days (see methods). In total we simulated tracks of 60,000 particles (30,000 driven by shallow currents, and 30,000 driven by deep currents) all with the same distribution of starting points based on known distribution of adults. The dispersal kernels for larvae from the three archipelagos differed, showing spatial variation in the ability of deep ocean currents to transport larvae (see Supporting Information S1). As expected, the deeper currents were generally slower and less effective at dispersing the larval particles. The exception to this is between Vanuatu and New Caledonia, where the deeper currents bring many more particles towards the New Caledonian populations, although this dispersal requires more than 250 days (see Supporting Information S1).

**Figure 2:**
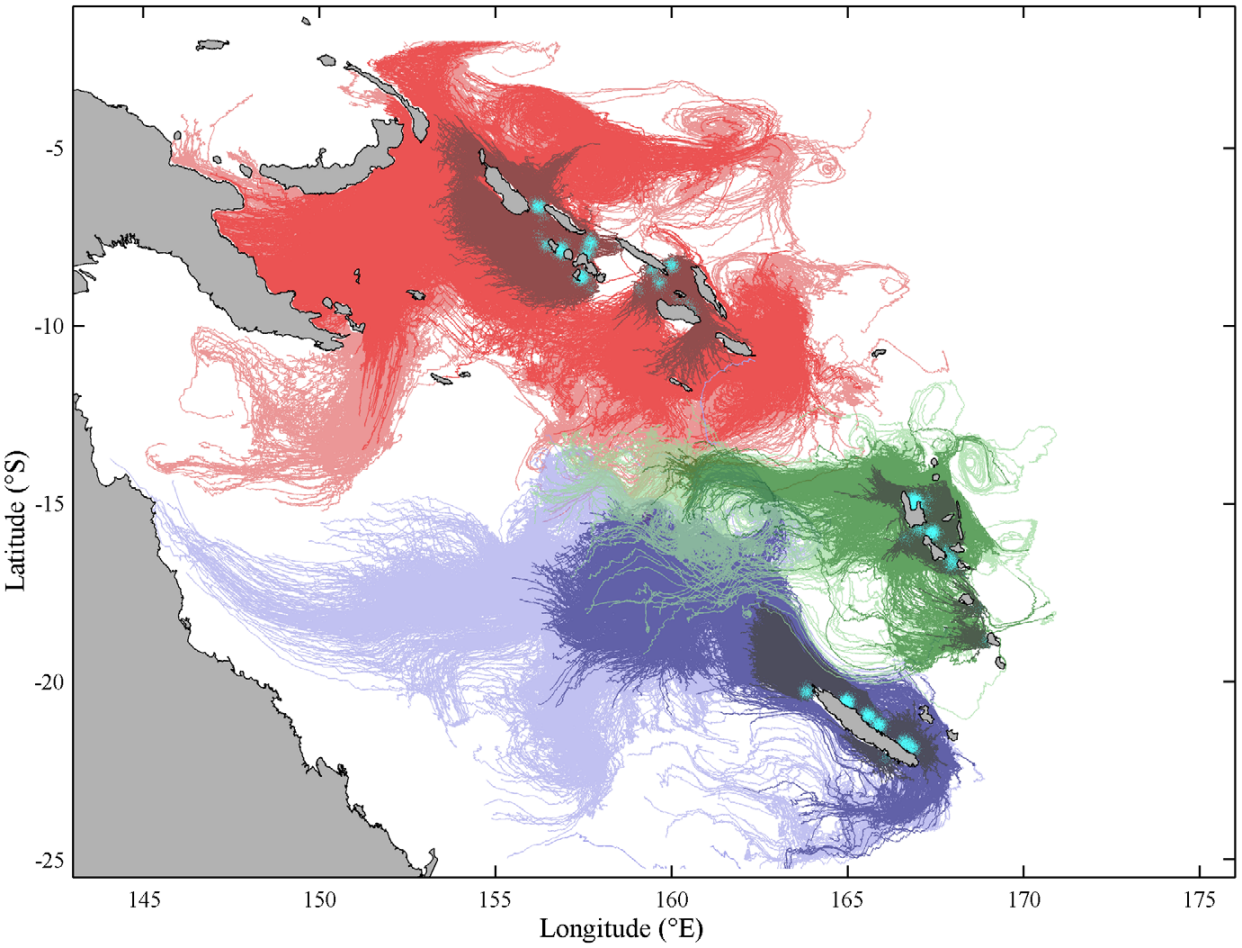
Particle tracks of 30,000 simulated larvae. The Solomon Islands, Vanuatu and New Caledonia each have 10,000 particle tracks (red, green and blue tracks respectively). A cyan dot marks the starting location of each particle. The tracks for 50, 250 and 500 days are shown in progressively lighter colours.

Considering just the distances between archipelagos (Table 2), and ignoring direction for the time being, we found that 250 days of dispersal in ocean currents between 800 m and 1400 m deep is generally sufficient to connect chiton localities within each archipelago, and thus support local retention (Table 2, Fig. 2, Supporting Information S2). However, inter-archipelago dispersal was only likely between New Caledonia and Vanuatu, whilst direct dispersal between the other archipelagos appeared to be extremely unlikely even after 500 days. Results for the deeper ocean currents showed a reduced larval dispersal potential (Supporting Information S1). Considering dispersal direction as well as distance (Fig. 2, Supporting Information S2) shows that the majority of larvae tend to drift westwards, away from the archipelagos, meaning that the connectivity between New Caledonia and Vanuatu is weaker than the naive expectation from the dispersal distances alone (Table 2, Fig. 2, Supporting Information S2).

**Figure 3:**
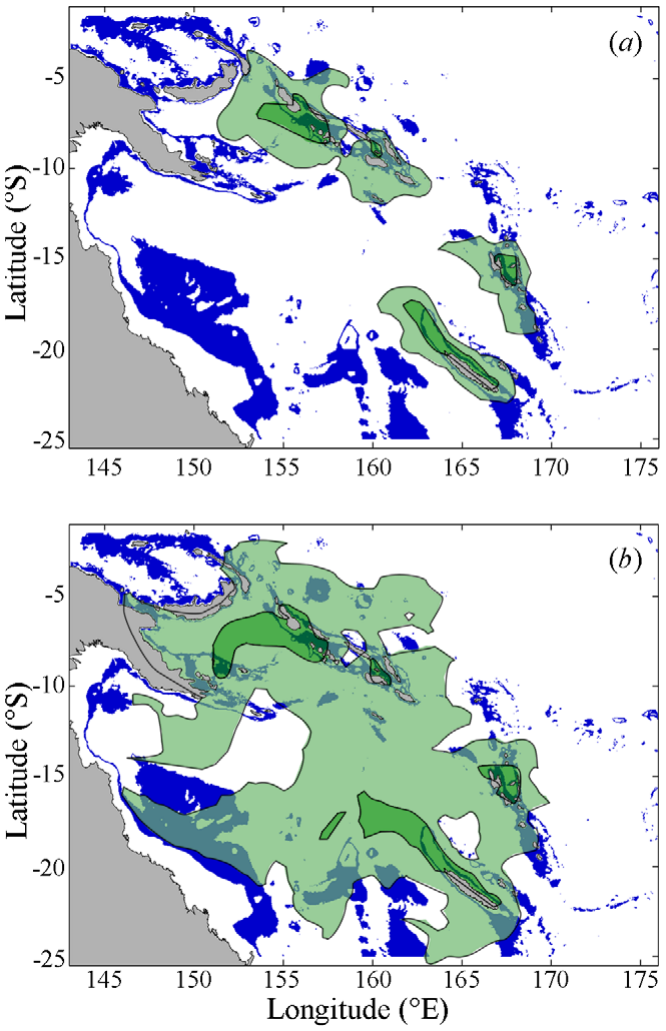
Particle distribution after 100 and 500 days and bathymetry between 200 m and 1600 m. The depths between 200 m and 1600 m within our study region (shown in blue) are consistent with the depth range of the observed chiton populations. Dark and light green regions show the probability that a randomly chosen particle from one archipelago crosses this boundary within (a) 100 days and (b) 500 days. Dark and light green regions represent a probability of 0.1 and 0.001 respectively.

We used our simulations to suggest regions that may contain as yet undiscovered populations of deep-sea chitons by combining the simulated dispersal plumes with the depth range of the known deep-sea chiton locations (Fig. 3). Within 100 days 10% of larvae from our New Caledonia locations could have reached the d'Entrecasteaux Reefs, 190 km north of New Caledonia (Fig. 3a). Similar accessible habitats were also found around Vanuatu and the Solomon Islands (e.g. Rennell Island). Considering 500 days of dispersal suggested habitats that could be reached in several generations for non-feeding larvae (Fig. 3b). This suggested the possibility of an extensive region of population mixing to the west of Vanuatu. The distribution of deep-sea chiton populations may be underestimated at present by an order of magnitude (separation distances of 150 vs 1500 km).

## Discussion

The discontinuity between known populations of deep-sea chitons is clear: paths originating from one archipelago do not reach any other archipelago within the restrictions of expected larval lifespan. The overlap at the longest extent of our simulations (500 days; i.e. multiple generations) agrees with faunal survey results, that most overlap is between the Solomon Islands and Vanuatu. Our results indicate a strong likelihood of intermediate, but as yet undiscovered, populations that could maintain species connectivity.

Our aim was to combine all the available data on our system: adult distributions from deep-sea survey data, larval life history inferred from shallow water and phylogenetically proximate analogues, and ocean currents inferred from autonomous profiling floats. All of these data are in themselves patchy and limited, but in combination they generate testable quantitative hypotheses that should focus the design of future studies of deep-sea biota. Primarily, we intended to test the viability of the null hypothesis that known populations in these three archipelagos can be connected by direct settlement of larvae. In order to explore the strengths and weaknesses of this approach to studying the dispersal of deep-sea benthic species, we consider a range of issues that may impact our model results, including physical and biotic factors.

An alternative approach, which can generate detailed, 3D current flow fields, is to use a hydrodynamic model that is driven by bathymetry data and surface forces, such as wind velocity (e.g. [27]) or to combine low resolution, hydrodynamic models of an ocean's general circulation with data such as the Argo array [28]. However, this approach is computationally and technically difficult to apply across an entire ocean basin at the fine resolution required to study long-distance dispersal. By contrast, our method is portable, and easily applied to new organisms and new ocean systems.

Overall our results demonstrate a strong mismatch between the observed species distributions (four species that range across the entire region) and the oceanographic currents that prevent larvae from travelling directly between known populations. This mismatch has at least three potential biotic explanations: (i) the larvae have a much longer PLD than predicted by available observations or models, (ii) the larvae make a significant vertical migration to exploit faster-moving surface currents and/or (iii) there are a large number of intermediate populations which provide bridges between the known centres. We consider each of these potential explanations below.

Dispersal time could hypothetically be lengthened by drifting as eggs, but eggs are probably only viable for less than 40 hours [29]. Fertilised eggs may persist for up to nine weeks before hatching in polar waters [30]; this additional drifting time is not included in most of the PLD data that are reported in other literature [21], [31] or modelled [12]. Polar regions are much colder than our tropical deep-sea localities (Fig. 1), but this could potentially add an additional 50-day interval to our PLD estimates. This upper limit still indicates that our simulations of 500 days represent multiple generations.

Deep-sea larvae may be longer lived than their shallow water counterparts, because the colder water temperatures of the deep-sea will lower metabolic rates [11], [32], [33]. But these tropical species are in water still subject to solar warming (Fig. 1). The longest proposed PLD for deep-sea taxa is approximately 13 months (400 days). This figure is based upon the observed delay between mussel spawning and settlement around a hydrothermal vent [5] and the longevity of asteroid larvae maintained in a laboratory [24]. In the former case, the larvae are known to have a veliger form, which is capable of swimming and feeding [5]. Longer PLDs, on the scale of 500 days, may well be within the capacity of feeding larvae of other animal groups. Lecithotrophic larvae may be capable of delaying metamorphosis in the absence of suitable habitat. Some larvae that lack a feeding mouth can still absorb environmental nutrients, which could prolong their dispersal potential [34]. Larvae are clearly not completely passive, but are unlikely to be able to extend their PLD beyond 400 days, which is the upper prediction from the model of O'Connor et al [12]. Long PLDs may also lead to an increased larval mortality (e.g. by prolonging exposure to predators). For this reason the PLD of cold-water species may be even lower than the predictions from temperature models [12]. The basic rate of larval mortality, estimated to be 60–90% [14], represents another significant barrier to connectivity that, if included in our model, would actually reduce predicted larval dispersal.

Trochophore larvae do not have the capacity to influence their migration by directed swimming, but may exhibit directed buoyancy or limited vertical migration. Some marine invertebrates (e.g. copepods) are known to deliberately exploit the difference in surface and deeper current speeds [35], [36]. There is a suggestion that some planktotrophic larvae of deep sea species exploit faster near-surface currents to increase dispersal [37], [38], [39], although warmer surface waters would also reduce expected PLDs. Chiton larvae may be able to move vertically up to 13.8 cm per minute [40], although it is unclear whether a non-feeding larva could maintain this speed for long enough to reach surface currents (40 hours from a starting depth of 300 m assuming no drift). Taking a global average, surface currents are estimated to be four to five times faster than deep-sea currents [41], but their PLD will also decrease by a factor of four to five, due to higher water temperatures (Fig. 1). Furthermore, the complex trajectories experienced by a particle could still prevent connectivity between the populations studied and add to the total larval mortality. Larvae that migrate into shallow water (perhaps in response to light cues, at depths less than 200 m) may increase their chances of colonising sunken wood before it has reached the deep-sea, although this has never been observed on floating wood substrates.

In this simplified model we have assumed that the larval particles are travelling within the depth range of the adult chitons that were collected in the field. This depth range will be a variable distance above the seafloor, due to changes in bathymetry across our study region (Fig. 3). The spatial and temporal averaging of the Argo probe data does not permit us to investigate the impacts of small-scale changes in current velocities, particularly in relation to local proximity of the seabed in different cells. These effects would have a large influence on the trajectory of individual larvae in real life but increased complexity of the trajectory, or slower currents next to the seabed, would make it even more difficult for larvae to traverse between our study populations.

There is no data to suggest seasonal spawning in chitons or aplacophoran molluscs in the deep sea [4]. Our data show some differences in current velocities across seasons (Supporting Information S1); one predictive outcome of this study is that we expect spawning in chitons and other benthic invertebrates at these depths to correlate with the fastest seasonal currents.

Extreme events, such as benthic storms [42], are unlikely to be captured by our oceanographic data, and could produce currents that are up to 25 cm/s for several days. For such extreme events to be an important driver of broad-scale population connectivity, they would need to be widespread throughout the south-west Pacific, the period between events would need to be less than the lifetime of a sunken wood substrate, and the chiton species would need to synchronise their reproduction with these unpredictable events. Such events will contribute to maintaining the observed evidence for connectivity but our model demonstrates system behaviour under normal conditions.

We conclude that the distributions of deep-sea chiton species observed in the south-west Pacific implies the existence of intermediate populations which provide stepping stones for larval transport to link the sampled areas in the Solomon Islands, Vanuatu, and New Caledonia. We have considered a range of alternative hypotheses and thought experiments to examine the impacts of the assumptions made in constructing this mathematical model. While some issues may influence the real distribution of benthic polyplacophorans in the study area, including successful local retention, the model results show clear evidence that direct larval transport between the known conspecific populations is unlikely. All scenarios to achieve contact between the three archipelagos are less parsimonious. Our predicted upper range for larval dispersal distances of 10^2^–10^3^ km (Table 2) is significantly smaller than the recent predictions from McClain and Hardy [43] of 10^2^–10^5^ km based upon constant unidirectional surface current velocities. This difference can be explained by the lack of evidence for unidirectional currents in the Argo data and the 5-fold difference in average current speeds between the surface and depths of 1000 m.

The results of this modelling approach provide concrete, testable hypotheses that can be validated with field work. Collecting biological data from the deep sea benthos is difficult and expensive, and this modelling approach presents a clear way to refine the field approach to maximise the efficacy of collecting efforts. In particular we can identify sites for the most likely intermediate habitats for the model species.

The deep-sea benthos remains the most inaccessible and least understood habitat on earth. The use of Argo oceanographic probe data is highly relevant to a wide range of marine dispersal questions but this study represents the first application for benthic communities. Argo probe data is a particularly rich resource that has been under-used for biological applications [44]. One major benefit of this approach is that it generates testable predictions at large, as well as finer scales. We predict that chitons and organisms with similar larval life history in the south-west Pacific areas will have a high degree of population genetic structure and that the observed connectivity is dependent on intermediate populations. At a finer scale, bathymetric data and probability maps indicate specific likely localities of those addition undiscovered chiton populations, such as near Rennell Island. It is unclear what mass of plant matter would be required to suffice as a stepping-stone habitat; frequent small deposits could be biologically effective but difficult for biologists to detect. Modern oceanographic recording data such as the Argo array, paired with quantitative modelling approaches, represent a major resource for studying ocean basin dynamics as well as the coastal applications that have been used prior to this study. All of these data are essential for understanding and conserving the biodiversity of the deep ocean.

## Methods

### Observed (starting) distribution of deep-sea chitons

The sources of larval particles were defined to be the recorded distribution of 1070 individual deep-sea chitons, representing 18 species, across the archipelagos of New Caledonia (17 locations), Vanuatu (50 locations) and the Solomon Islands (56 locations) [17], [19], [45].

We preferred to use the largest possible set of larval source locations in order to minimise the possibility of underestimating population connectivity. Therefore the oceanographic data used for our main simulations is from depths of 800–1400 m, whilst the total chiton dataset includes records from 200–1600 m. We also completed dispersal simulations for a limited number of localities corresponding to a single taxon, *Nierstraszella*. These results supported the use of an expanded starting distribution drawn from the locations of all recorded adults. Using the largest possible set of larval source locations does not imply mixing between taxa.

### Estimating deep-sea oceanographic currents

Deep-sea ocean currents were estimated using data from the global Argo array [25]. To achieve sufficient data coverage across our study region we amalgamated data across depths, months and years. This means that temporal covariances in the current data have been removed from our simulations. These omissions are validated to a first order of approximation by the fact that the main source of variability in the ocean current data is between different latitude-longitude positions, and serial autocorrelations in the probe data have no detectable effect (Supporting Information S1).

Argo probes repeatedly perform a cycle of dive-drift-resurface-transmit data. Each probe is programmed to dive down to a predefined pressure and stay at this pressure for roughly 10 days before resurfacing and broadcasting its position and other data back, via satellite, to a data centre. When data transmission is complete, the probe dives once more and starts another cycle. The Argo probe data for our study region from January 2001 until May 2009 were downloaded from the Coriolis Data Centre (http://www.coriolis.eu.org).

Initial statistics from the data are shown in Supporting Information S1. For the primary analysis, we selected probe data in two partitions: depths of 800 m and 1400 m (4915 cycles in our region of interest), and 1400 m–2500 m deep (4723 cycles). We excluded the data from 13 cycles below 2500 m as not relevant to the model organism; and also discarded the 225 cycles that were shallower than 800 m since we were uncertain of the quality of these data. The results we derive in this paper use the more shallow data partition (800 m–1400 m); results of comparative analysis with currents modelled from the deeper partition (1400 m–2500 m) are presented in Supporting Information S1.

#### Simulating trajectories from an Eulerian vector field

We divided the rectangular region 143°W–176°W, 1.5°S–25°S into a 0.1°×0.1° grid. At each intercept of this grid we defined a velocity, **u** = (u_1_, u_2_), in units of degrees per day (see below for details on how these vector fields were constructed). The drift of a particle at position longitude r_1_ and latitude r_2_ in this vector field was then described by
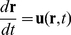
(1)where **r** = (r_1_, r_2_) and **u**(**r**, *t*) is the velocity at point **r** at time *t*, which was calculated by a cubic interpolation of each velocity component u_1_ and u_2_ from the gridded data. Equation (1) was integrated using an explicit Runge-Kutta (2,3) method to give the trajectory of a particle in the vector field, **r**(t).

#### Reconstructing Eulerian velocity fields from Argo probe data

For the nth cycle, the Argo data gave us estimates for the position at which the probe dived, **x** ^o^(n) and resurfaced **y** ^o^(n), and the time between these two events, t(n). The vectors **x** ^o^(n) and **y** ^o^(n) each have two elements containing the longitude and latitude of a location. With these data we calculated a raw velocity vector for the nth cycle (**v**(n), **X**(n)), where **v**(n) = (**x** ^o^(n)−**y** ^o^(n))/t(n) is the velocity and **X**(n) is the position of this vector, which we chose to be the mid-point location of the cycle, **X**(n) = (**x** ^o^(n)+**y** ^o^(n))/2. The Argo data give us no information about the vertical component of the ocean currents, but by comparing simulations from two depth ranges we can assess the uncertainty that vertical movements would have upon our results.

To reconstruct Eulerian velocity fields from the Argo probe data we used an optimal interpolation method [26]. This method uses a zeroth order assimilation formula, based upon a Kalman filter, to iteratively correct an initial velocity field estimate using the Argo data. The method required an initial estimate for the Eulerian vector field at the intersections of our 0.1°×0.1° grid, **u** ^b^. This vector field was then used in equation 1 to predict the final point of the nth cycle, **y** ^b^(n). From this we calculated a model estimate for the probe velocity during the nth cycle, **v** ^b^(n) = (**y** ^b^(n)−**x** ^o^(n))/t(n). We then calculated a new vector field, **u** ^a^, which was the vector field **u** ^b^ with a correction factor to incorporate the information from the Argo probes
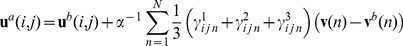
(2)where is the corrected vector field at the (*i*, *j*) th grid intersection, *α* ^−1^ = 1+σ_o_^2^/σ_b_^2^, σ_o_/σ_b_ is the error of the observed probe locations relative to the simulated trajectories, *N* is the total number of cycles that are being used from the data and *γ^1^_i j n_*, *γ^2^_i j n_*, *γ^3^_i j n_* are weights. These weights were defined as
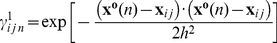
(3a)

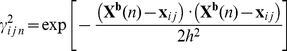
(3b)

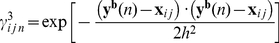
(3c)where *h* = 0.1 is the spacing of the grid and **x**_ij_ is the location of the (i, j) th grid intersection, **X** ^b^(n) = (**y** ^b^(n)+**x** ^o^(n))/2 and • represents the vector product. These three weightings corrected the vector field **u** ^b^ near the start, **x** ^o^(n), mid-point, **X** ^b^(n), and end, **y** ^b^(n) of each simulated trajectory. Since the Argo probe data gave us no information about a probes trajectory whilst it was submerged, but our particle simulations must recreate these trajectories, we took σ_o_/σ_b_ to be 0.1, so that the Argo locations, **y** ^o^(n), were an order of magnitude more precise than our simulated locations, **y** ^b^(n). We iterated this correction procedure three times, each time setting our estimated vector field **u** ^b^ to be the corrected vector field from the last iteration (i.e. setting **u** ^b^ = **u** ^a^).

Rather than using every available piece of probe data to reconstruct a single vector field, we created 500 randomly thinned subsets of our probe data. This was done by selecting every fourth grid square (so that 1/16 of the squares on the grid were selected) and from each of these grid squares randomly picking one probe cycle whose mid-point, **X**(n), lay within the grid square. This data thinning procedure has two advantages over using the whole dataset. Firstly, the 500 random data subsets capture some of the variability in the data. If we had used all the data to produce one vector field, this vector field would have been an average of nine years' of data, with no information about the variability across these nine years. Secondly, two probes in the same vicinity can have conflicting trajectories because nine years of data have been amalgamated. These conflicts make it impossible to meaningfully describe the trajectories of all probes with one vector field, whereas our thinned data rarely suffers from these conflicts.

For each thinned dataset we made an initial estimate of the Eulerian velocity field, **u** ^b^, by interpolating the two components of the probe velocities, **v**(n), with a triangle based cubic interpolator [46], to give values for the velocity components, **u** ^b^(*i*, *j*), at the (i, j) th intersection of the grid. We could have used other interpolation approaches, and none will give exactly the same answer (e.g. writing the velocity components in polar coordinates will affect the interpolation results), but the interpolated vector field is sufficiently accurate as an initial estimate. This initial estimate, **u** ^b^, was then used to start the optimal interpolation described above. The final interpolated vector fields allowed us to predict the probe locations **y** ^o^(n) to within a median error of 0.01°.

#### Correcting the vector fields for the presence of land

Our interpolated vector fields, **u** ^a^, did not incorporate information about the land. In some cases where the land drops steeply into the ocean, the probes could pass close to the coastline and the interpolated vector fields naturally followed the deep-coastal currents. However, where probe data were sparse (e.g. where the ocean was too shallow) the interpolated vector field could give ocean currents that crossed land. To avoid particle paths crossing large areas of land we made simple modifications to the vector field. Coastline data for our region were downloaded from National Geophysical Data Centre (http://rimmer.ngdc.noaa.gov). We identified all vectors in **u** ^a^ that were over land, adjacent to sea and whose velocity vector pointed toward land, and conversely also vectors that were over the sea, adjacent to land and whose velocity vector pointed toward the land. We then rotated these vectors through the smallest possible angle that would make them point towards the sea.

### Estimating planktonic larval duration

We compiled published planktonic larval duration data [12], [21] using approximate seasonal average temperatures for the range of each species where it was not reported. We removed the three outlier species (*Laqueus californianus*, *Limulus polyphemus*, and *Callianassa tyrrhena*). We then fitted the exponential-quadratic mixed model proposed by O'Connor *et al.*,
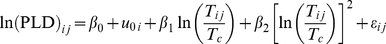
(4)where ln(PLD)_ij_ is the natural logarithm of the planktonic larval duration for the j^th^ record of the i^th^ species, T_ij_ is the water temperature for the data point ln(PLD)_ij_, T_c_ = 15°C, *u_0i_*∼*N(0,τ^2^)* is a random intercept following a normal distribution with zero mean and variance *τ* ^2^ for the i^th^ species and *ε_ij_*∼*N(0,σ^2^)* is a normally distributed error term with zero mean and variance *σ* ^2^. The nlme package in R [47] was used to fit the model. Our generated parameter estimates, *β_0_* (2.95±0.11 s.e.), *β_1_* (−1.30±0.06 s.e.), *β_2_* (−0.26±0.05 s.e.), and the two variances *τ* ^2^ (0.92) and *σ* ^2^ (0.03), were then used to predict PLDs for our deep-sea chitons.

Water temperatures for the recorded locations of deep-sea chitons were downloaded from the USGODAE Live Access Server (http://www.usgodae.org/las/getUI.do) using the “Navy GDEM Monthly Temperature/Salinity/Sound Speed” dataset, by downloading individual temperature records for the approximate date, coordinates, and depth of the collecting locations of the adult chiton samples. We used the minimum temperature (i.e. maximum depth) for the range of sampling depths at a given location. Since chiton larvae from shallow water species are observed to be lecithotrophic, we assume that this will be the case for our deep-water species. We therefore calculated the Best Linear Unbiased Predictor (BLUP) of the intercept for each lecithotrophic species in our data set. We then selected the species with the largest BLUP (i.e. *Echinaster* Type I [48]), which gave an intercept of 3.54. This intercept was used to predict the PLDs based on the temperatures for the 107 recording locations. The equation relating our best estimate of PLD (days) with temperature (°C) is
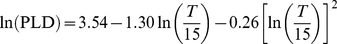
(5)where T is the minimum water temperature at a recording location. Confidence intervals on these predicted PLDs were calculated by simulating the model with both random error terms and calculating the 2.5% and 97.5% percentiles of the PLD distribution (Fig. 1).

### Simulating the dispersal pathways

The starting location of each path was drawn from a 2-dimensional Gaussian probability distribution with standard deviation 0.1° and centred on a recorded chiton location. This known location (a sample station from the original collecting cruise) was selected in proportion to the abundance of sampled individuals relative to the other known locations of chitons. To include the variability inherent in the Argo data we selected 5 vector fields for each simulated path, and for each day of the simulation randomly used one of these 5 vector fields.

The method employed is a novel combination of multiple data sources which have never previously been applied to questions of the dispersal of deep sea benthic organisms. We further present these methods and the data from our chiton test case as Matlab files [49] that can be modified for any application using the worldwide Argo data (Supporting Information S3, S4, S5, S6, S7, S8, S9). These files contain: the MNHN survey data for deep-sea chitons (Supporting Information S3), the Matlab script to simulate larval dispersal (Supporting Information S4), the Matlab script to calculate rate of latitude and longitude from ocean current data (Supporting Information S5), the Matlab script to generate ocean current vector fields from Argo probe data (Supporting Information S6), the Matlab script to read the Argo data netCDF files from the Coriolis web server http://www.coriolis.eu.org/cdc/argo.htm (Supporting Information S7), data for the coastline in our study region (Supporting Information S8) and the Argo probe data used in this paper (Supporting Information S9).

## Supporting Information

Supporting information S1 **Interrogation of the ARGO probe data [PDF].** Results of analyses to demonstrate limited variability over seasons, depth partitions, and multi-year periods in the nine year dataset of oceanographic current data.
(PDF)

Supporting information S2 **Dispersal pathways [animated GIF].** Animation demonstrating particle (predicted larval) pathways originating at the source populations of the model organism, chitons.
(GIF)

Supporting information S3 **Deep-sea chiton dataset [XLS].** An Excel file that contains the abundance (number of specimens) and distribution of deep sea chiton species. These data encompass several species of chiton because the majority of simulations in the paper used these data. Data upon individual chiton species can be obtained from the authors.
(XLS)

Supporting information S4 **simulateParticles.m [Matlab script].** This Matlab script performs particle tracking using the vector fields generated by generateVectorFields.m (Supporting Information S3) to drive the simulation, and the observed chiton distribution contained in Supporting Information S3 to initialise the positions of the particles.
(MAT)

Supporting information S5 **dlongdlat.m [Matlab script].** This Matlab script calculates a particles rate of change of position (longitude and latitude in degrees per day) from the ocean current vector fields and particle's position.
(MAT)

Supporting information S6 **generateVectorFields.m [Matlab script].** This Matlab script uses the Argo probe data in probeData.mat (Supporting Information S9) to estimate deep sea ocean currents. It will generate several files, each using a random subset of the data to estimate the ocean currents.
(MAT)

Supporting information S7 **readArgoData.m [Matlab script].** This Matlab Script reads the NETCDF files that contain Argo probe data from the Coriolis web sever http://www.coriolis.eu.org/cdc/argo.htm. The output from the script is contain in Supporting Information S9.
(MAT)

Supporting information S8 **coastline.mat [Matlab binary file].** This Matlab binary file (.mat) contains the coastline data for the islands in our study region.
(MAT)

Supporting information S9 **probeData.mat [Matlab binary file].** This Matlab binary file (.mat) contains the Argo probe data used in the paper. This file is used by generateVectorFields.m (Supporting Information S6).
(MAT)
