# Supplementary material for: Larval Transport Modeling of Deep-Sea Invertebrates Can Aid the Search for Undiscovered Populations
Source: PLoS One. 2011 Aug 8;6(8):e23063. doi: 10.1371/journal.pone.0023063 (PMC3152551; doi:10.1371/journal.pone.0023063)

**Larval transport modelling of deep-sea invertebrates points to potential undiscovered populations**

Jon M. Yearsley (corresponding author)

University College Dublin, School of Biology and Environmental Science, Belfield, Dublin 4, Ireland, jon.yearsley@ucd.ie

Julia D. Sigwart

Queen’s University Belfast, School of Biological Sciences, Marine Laboratory, Portaferry, BT22 1PF, UK, j.sigwart@qub.ac.uk

**Supporting Information S1:**

Our overall aim was to simulate the dispersal pathways of our chiton larvae in the south-west Pacific, and from these pathways to estimate the dispersal distances of these chiton larvae, the connectivity of the locations where they are known to occur and suggest potential locations of, as yet, undiscovered populations. This was a test case to develop a novel method for using deep-sea oceanographic current data, to make inferences about the dispersal and distribution of deep sea benthic species. To do this we combined data on the distribution of deep-sea chitons from surveys, Argo autonomous profiling float data to reconstruct deep ocean currents, and chiton comparative physiology to predict limits upon the time a chiton larvae can survive in the water column. The methodology detailed below concerns exploratory modelling to test the viability of using Argo probe data for this type of research. All methods, unless otherwise stated, were implemented with Matlab.

***Argo Probes***

Argo probes repeatedly perform a cycle of dive-drift-resurface-transmit data. Each probe is programmed to dive down to a predefined pressure and stay at this pressure for roughly 10 days before resurfacing and broadcasting its position and other data back, via satellite, to a data centre. When data transmission is complete, the probe dives once more and starts another cycle. The Argo probe data for our study region from January 2001 until May 2009 were downloaded from the Coriolis Data Centre (http://www.coriolis.eu.org).

Initial statistics from the data are shown in Fig. S1-S5. For the primary analysis, we selected probe data in two partitions: depths of 800m and 1400m (4915 cycles in our region of interest, Fig. S1, S2), and 1400m–2500m deep (4723 cycles, Fig. S1, S2). We excluded the data from 13 cycles below 2500m as not relevant to the model organism; and also discarded the 225 cycles that were shallower than 800m since we were uncertain of the quality of these data.

***Seasonal and Yearly Variability in the Argo data***

The raw velocity data from the Argo probe data are well described by a gamma distribution, which we fitted to the data using the fitdistr function in the MASS package of statistical software R. Fitting a Gamma distribution to the ocean current velocity data from the Argo array gave a mean probe velocity of 3.74 km/day and a variance of 6.72 $(km/day)^2$ for probe data from depths between 800m and 1400m (Fig. S3.A). Data from the deeper probes, between 1400m and 2500m, gave a mean probe velocity of 3.53 km/day and a variance of 5.46 $(km/day)^2$ (Fig. S3.B)

To look at the effect of variability between seasons and variability between years we divided up our region into 2°x2° lat-long squares to remove some of the spatial variation from the data. We then divided up the data into the shallow and deep depth classes to remove some of the variation due to depth. Finally, we further divided the data into either three-monthly seasonal quarters across all years (Fig. S4), or sequential epochs containing two years of data (Fig. S5). Epoch 1 (2001-2003) was rejected from any further analyses because the data coverage was too sparse. Regions were selected with at least 5 probe cycles in any one quarter/epoch.

We tested the effect of spatial variation in ocean current velocities by randomly sampling (with replacement) the velocities of 30 probe cycles across all 2°x2° regions containing at least five probe cycles, and then repeating this procedure for each region individually. We then calculated the total displacement distance, assuming that each velocity vector acted on a drifting particle for one day. The resulting distances therefore approximate potential dispersal capabilities over 30 days assuming that spatial structure within each 2°x2° region is unimportant, or that spatial structure across the entire study area is unimportant. We repeated this 1000 times in order to generate distributions of dispersal distances (Fig. S6).

We used a very similar procedure to test for the effects of seasonal variation within each 2°x2° region by randomly selecting the velocity data from 30 cycles (with replacement) across all quarters, and then repeating this for each quarter individually (Fig. S7). The same approach was also used to test for the effect of ocean current variations between epochs 2, 3 and 4 (Fig. S8).

The results show that averaging across spatial regions (Fig. S6), seasons (Fig. S7), or epochs (Fig. S8) all lead to some systematic underestimation of dispersal

distances. This bias is more severe for our shallow depths where ocean currents are faster and more variable. But the largest bias is seen when we average across regions (Fig. S6). Our main results are based upon spatially explicit particle tracking simulations, so this bias does not enter into our results. We do average across seasons and across years, and so some bias is expected, but this bias is expected to be small compared to the spatial variation.

We further investigated the bias introduced by averaging across seasons by dividing the Argo data into the four quarters (Fig. S4) and performing our particle tracking simulations over 500 days with ocean currents that are estimated for each quarter independently. Data from all probes between 800m and 2500m deep were used to increase the spatial coverage of the data. Each particle simulation was started on a randomly chosen day during the year, and each quarter lasted for 91 days. Therefore, a particle starting on day 1 would experience currents from Quarter 1 for the first 91 days, followed by currents from Quarter 2 for the next 91 days, and then Quarter 3, Quarter 4, back to Quarter 1, etc. All particles had starting locations consistent with the relative distribution of deep-sea chitons around the archipelago of the Solomon Islands.

The dispersal distances of our simulated particles show a two-fold difference in median dispersal distance after 50 days, but this quickly decreases so that by 500 days the median dispersal distances show little effect of the starting quarter (Table S1). Comparison between these results and our original, season averaged simulations shows that the median dispersal distance is overestimated by our original simulations for particles from the Solomon Islands, contrary to our earlier expectations (Table S1, Fig. S9). The 2.5 and 97.5 percentiles of the dispersal distance distribution shows much less seasonal differences than the median, showing

that our 95% quantiles are robust to our averaging across seasons. The overall conclusion is that for the scale of our simulations, and with the present Argo data, averaging data across the quarters of the year has little effect upon our quantitative results.

Table S1: Median dispersal distances (in kilometres) by simulated larvae originating from the Solomon Islands and travelling for 500 days with the quarterly ocean currents estimated from probe data between 800m and 2500m deep. In the right-hand column are the results from Table 1 (main text), obtained by averaging across all quarters. Particles start on a random day of the year. The numbers of simulated particles starting in each quarter are 1375, 1360, 1415, 1380 in quarters 1-4 respectively. The 95% quantiles are shown in brackets. Initial larval distribution corresponds to the known distribution around the Solomon Islands.

| | Starting Quarter | | | | |
|---|---|---|---|---|---|
| | Quarter 1 | Quarter 2 | Quarter 3 | Quarter 4 | Averaged |
| 50 days | 69<br>(11 – 371) | 77<br>(12 – 394) | 87<br>(14 – 230) | 127<br>(22 – 298) | 162<br>(28 – 357) |
| 100 days | 131<br>(12 – 564) | 145<br>(23 – 454) | 189<br>(28 – 416) | 197<br>(23 – 434) | 285<br>(46 – 539) |
| 250 days | 332<br>(63 – 657) | 327<br>(84 – 648) | 306<br>(59 – 689) | 377<br>(32 – 687) | 489<br>(65 – 954) |
| 500 days | 428<br>(107 – 817) | 493<br>(202 – 839) | 549<br>(182 – 782) | 413<br>(127 – 801) | 560<br>(69 – 1159) |

Figure S1: The spatial distribution of the mid-point locations, X(n), of the Argo probes used in this study, illustrating the density of current data across the study region. Shaded rectangles give the bounding boxes of the chiton populations sampled around the Solomon Islands (northernmost), Vanuatu (central) and New Caledonia (southernmost), reported by Sirenko (2001) and Sigwart (2008). (a) Upper panel shows Argo probes between 800-1400m deep, (b) lower panel shows probes between 1400-2500m deep.

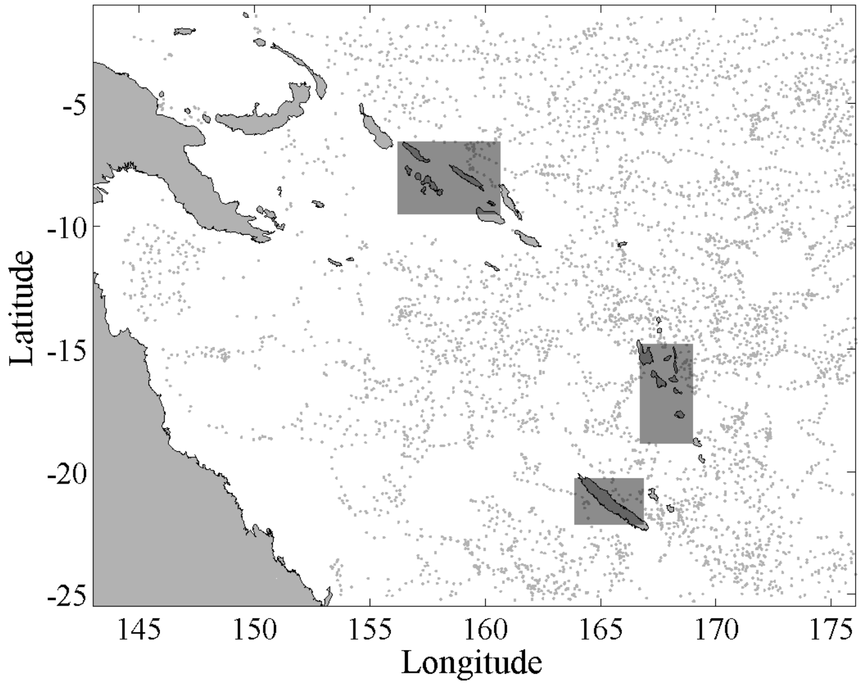


(a)

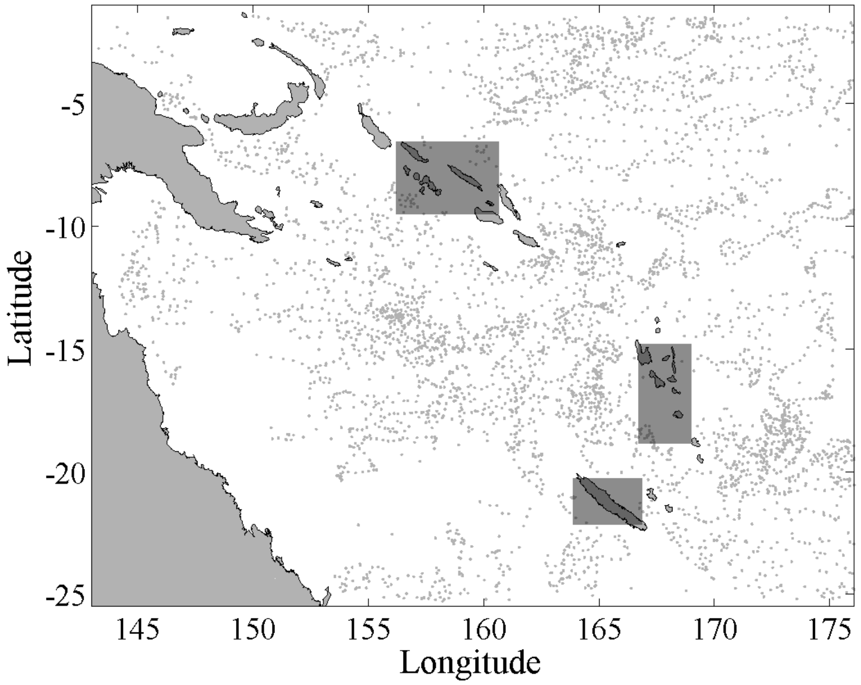


(b)

Figure S2: Histogram of the number of probe cycles in our study region between 2001 and 2009 against depth. The majority of cycles are between 1000m and 2000m deep.

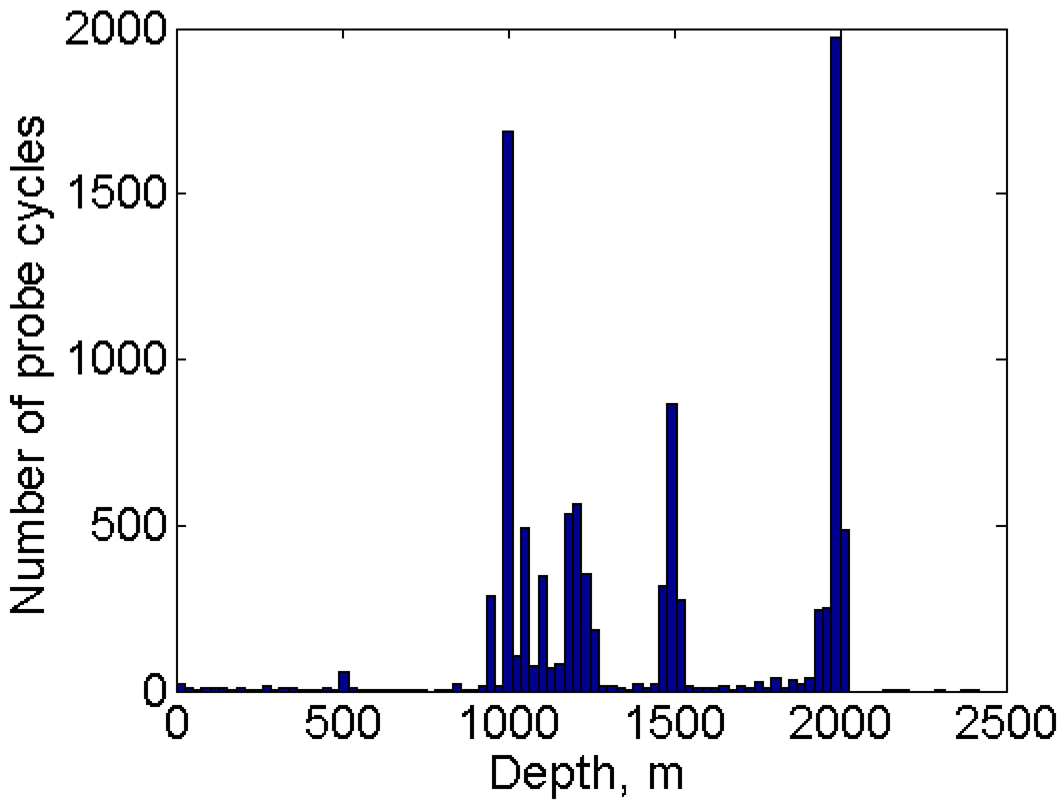

Fig S3: Distribution of probes velocities (solid line) for a) ‘shallow’ probes in the depth range 800-1400m and b) ‘deep’ probes in the depth range 1400-2500m. The best-fit gamma distribution (dotted line) has a mean, μ, and variance, $\sigma^2$, of μ=3.74 km/day and $\sigma^2$=6.72 (km/day)$^2$ for shallow probes, and μ= 3.53 km/day and $\sigma^2$=5.46 (km.day)$^2$ for deep probes.

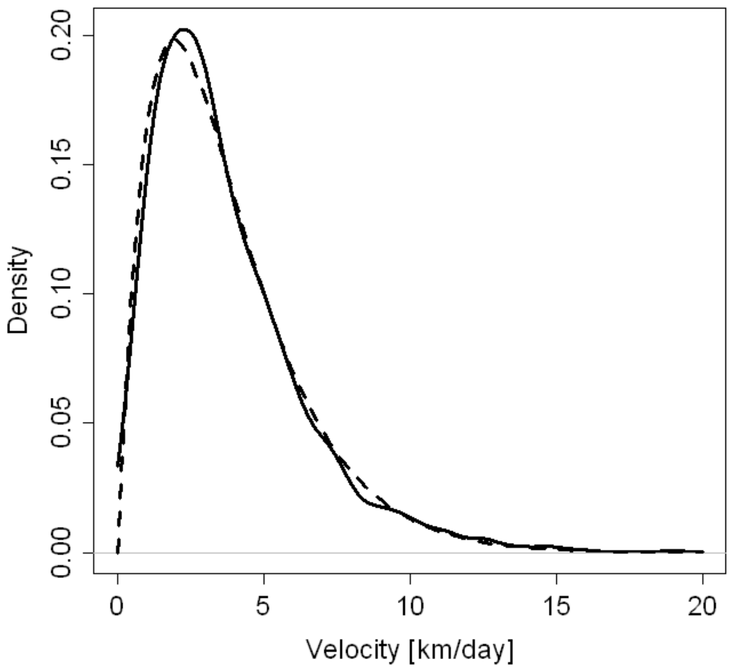


(a)

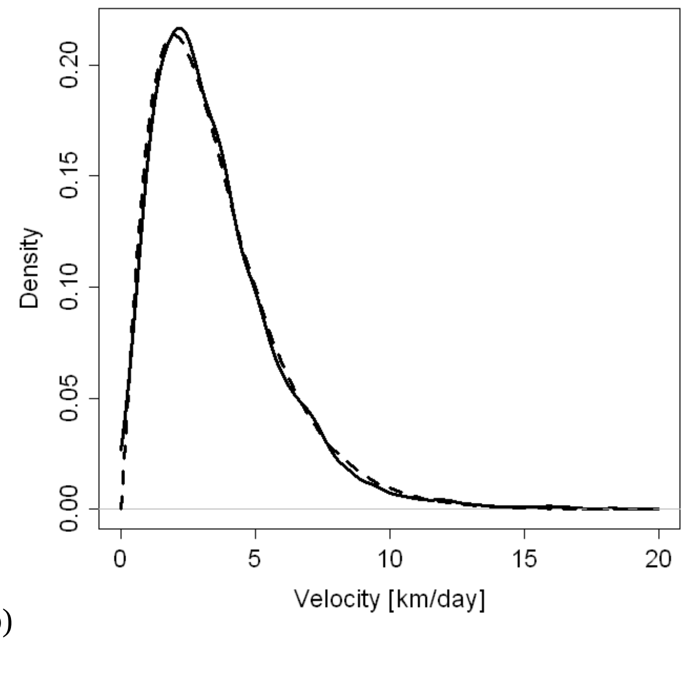


(b)

Fig S4: Distributions of mid-points of the probe cycles across the four quarters of the year for shallow (800-1400m, red dots) and deep (1400-2500m, blue dots). The 2°x2° lat-long grid is shown by the grey lines. Quarter 1, January-March; quarter 2, April-June; quarter 3, July-September; quarter 4, October-December.

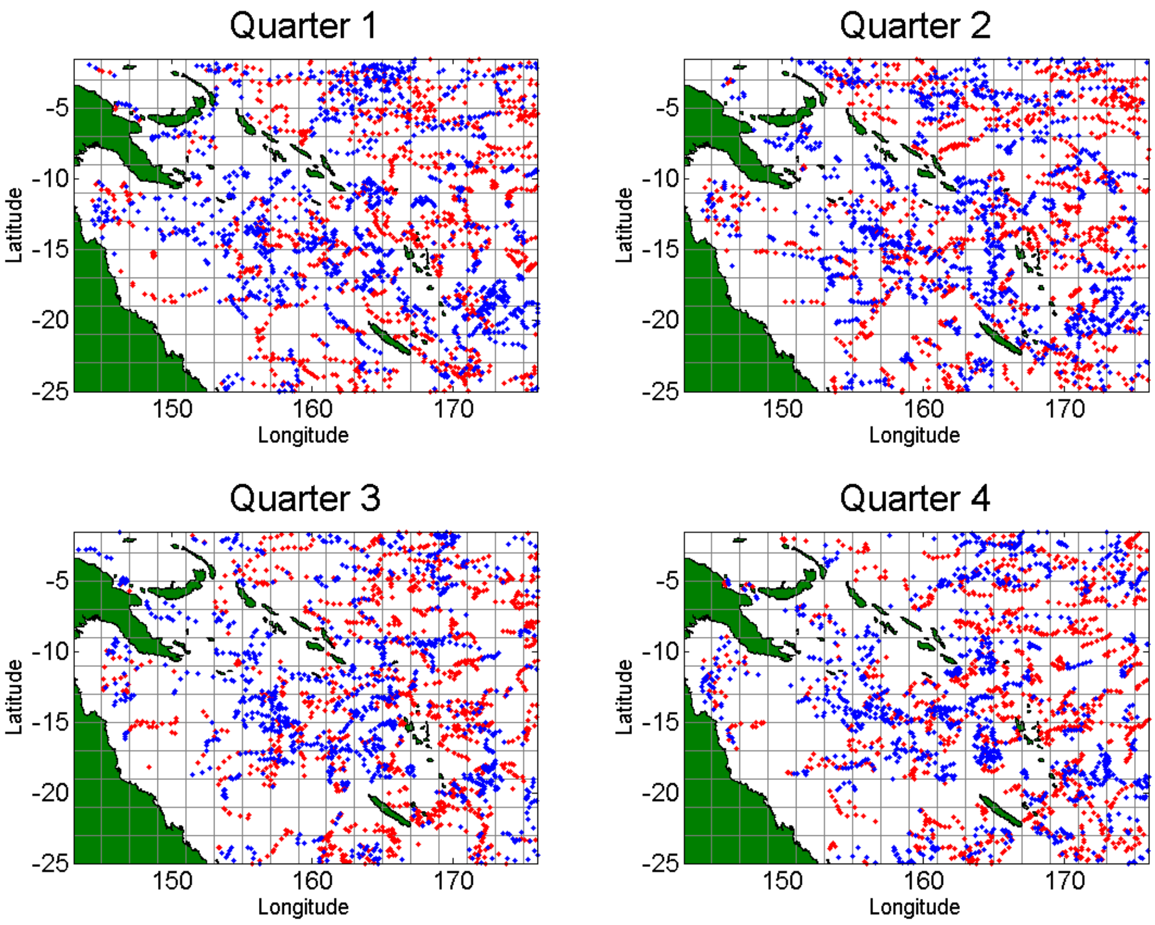

Fig S5: Distributions of the mid-points of the probe cycles across the four epochs (2001-2003, 2004-2005, 2006-2007 and 2008-2009) for shallow (800-1400m, red dots) and deep (1400-2500m, blue dots). The 2°x2° lat-long grid is shown by the grey lines. Epoch 1 (2001-2003) was removed from further analysis of variation, although those data were included in the total simulation vector fields.

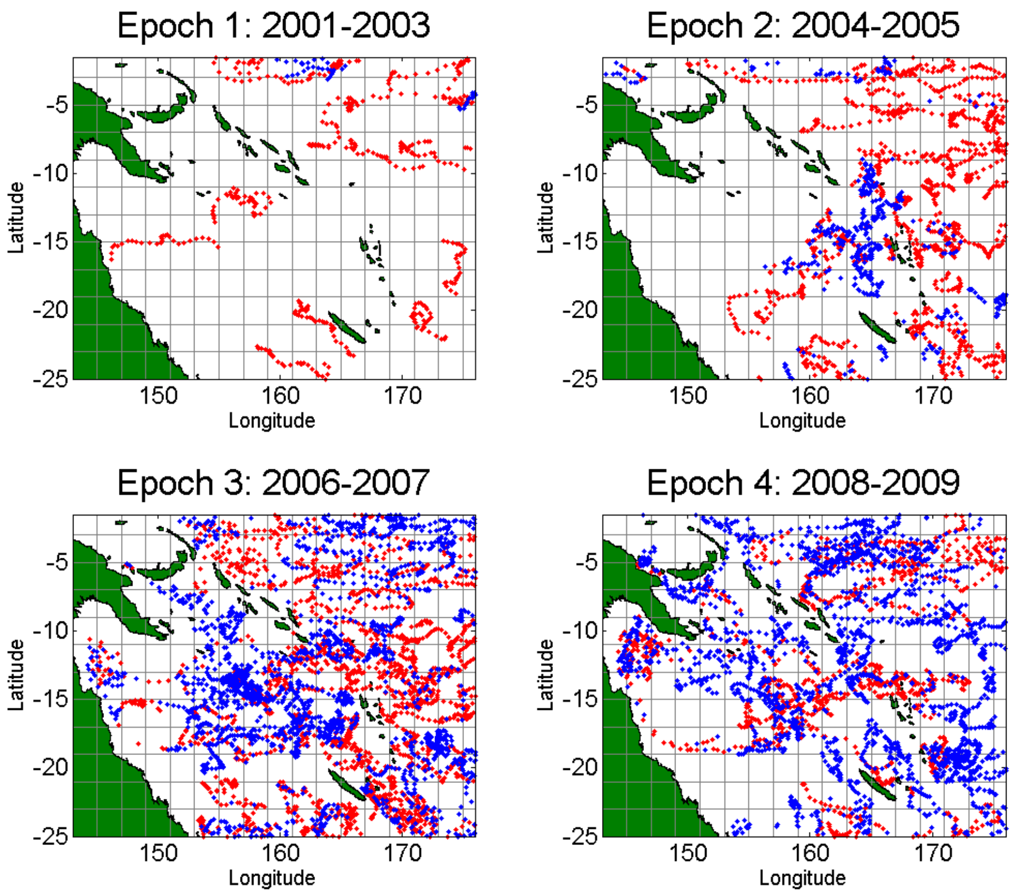

Figure S6: The distribution of simulated dispersal distances across 30 days for data from a) shallow probe cycles between depths of 800m and 1400m, b) deep probes cycles between depths of 1400m and 2500m. Each day, dispersal is inferred from the velocity of one probe cycle which is either randomly selected from data across all 2°x2° regions containing at least 5 probes cycles (black line), or randomly selected solely from one of these 2°x2° regions (grey lines). There are 156 and 157 regions that contain data on at least five shallow and deep probe cycles respectively. Each dispersal distribution is based upon 1000 distances.

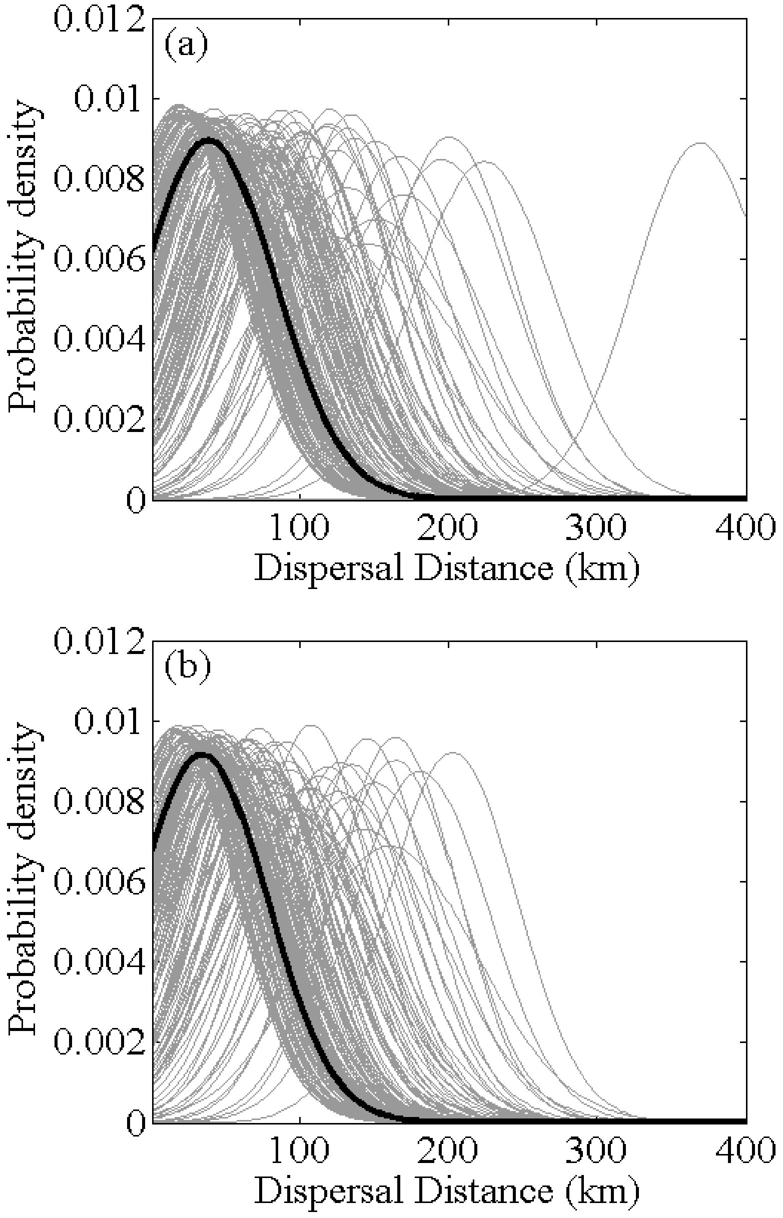

Figure S7: The distribution of simulated dispersal distances across 30 days for data from a) shallow probe cycles between depths of 800m and 1400m taken from 15 regions of 2°x2°, b) deep probes cycles between depths of 1400m and 2500m taken from 24 regions of 2°x2°. Only regions with sufficient data in all four quarters are used (figure 3). Each day dispersal is inferred from the velocity of one probe cycle which is either randomly selected from data across the entire year (black line), or randomly selected solely from one of the quarters (coloured lines). Each 2°x2° region has 1000 simulated dispersal distances.

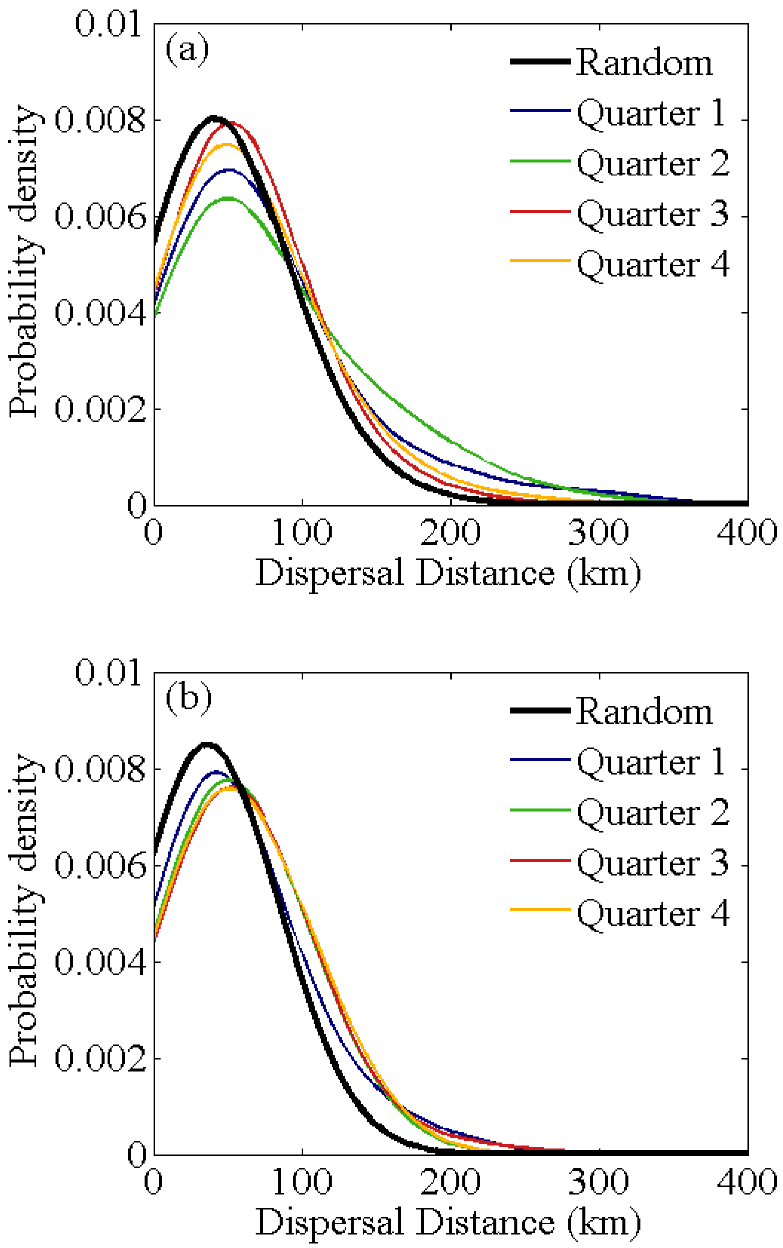

Figure S8: The distribution of simulated dispersal distances across 30 days for data from a) shallow probe cycles between 800m and 1400m deep taken from 10 regions of 2°x2°, b) deep probes cycles between 1400m and 2500m deep taken from 12 regions of 2°x2°. Only regions with sufficient data in all three periods are used (figure 3). Each day dispersal is inferred from the velocity of one probe cycle which is either randomly selected from data across all years between 2003 and 2009 (black line), or randomly selected solely from one two-year period (coloured lines). Each 2°x2° region has 1000 simulated dispersal distances.

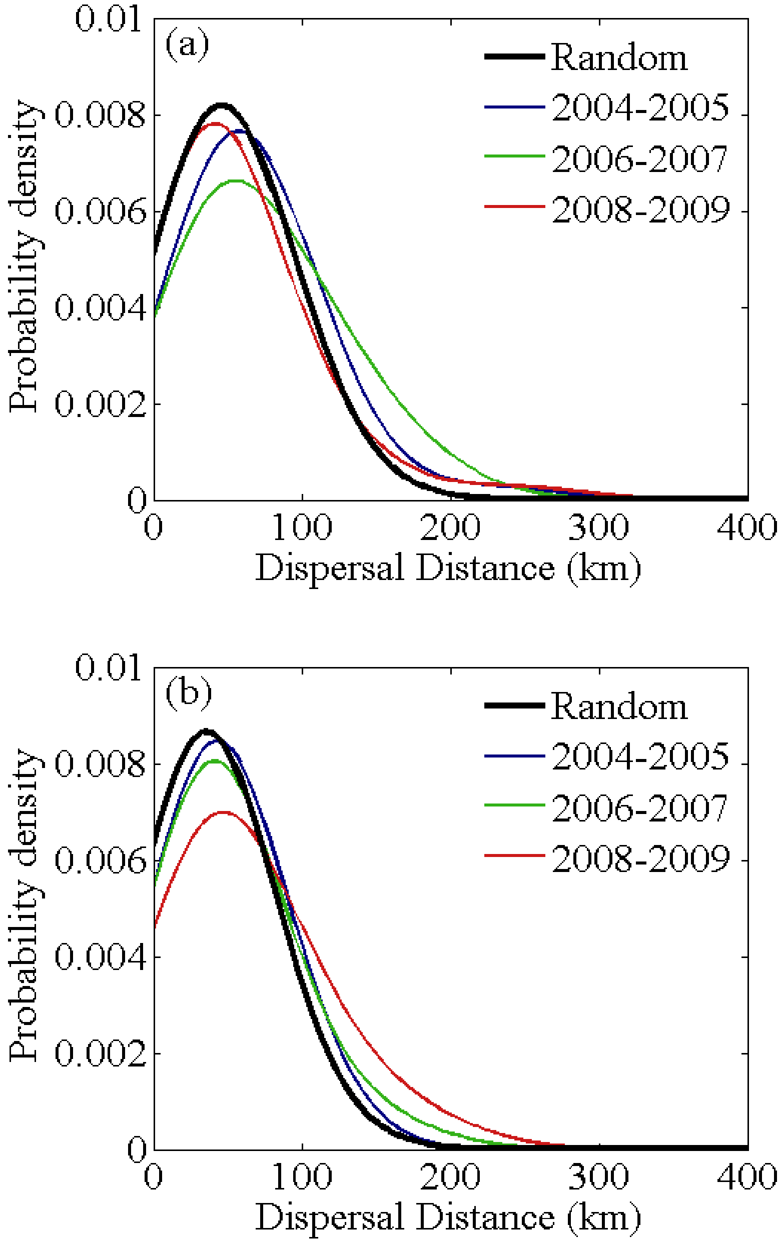

Figure S9: The dispersal kernels for particles from the Solomon Islands for simulations starting in a) Quarter 1 b) Quarter 2 c) Quarter 3 and d) Quarter 4. Particles are driven by ocean currents estimated for each quarter for the depth range 800-2500m. Solid, dashed, short-dashed and dotted lines show dispersal kernels after 50, 100, 250 and 500 days respectively. Curves obtained from a normal kernel smoothing density estimate with a bandwidth of 100km.

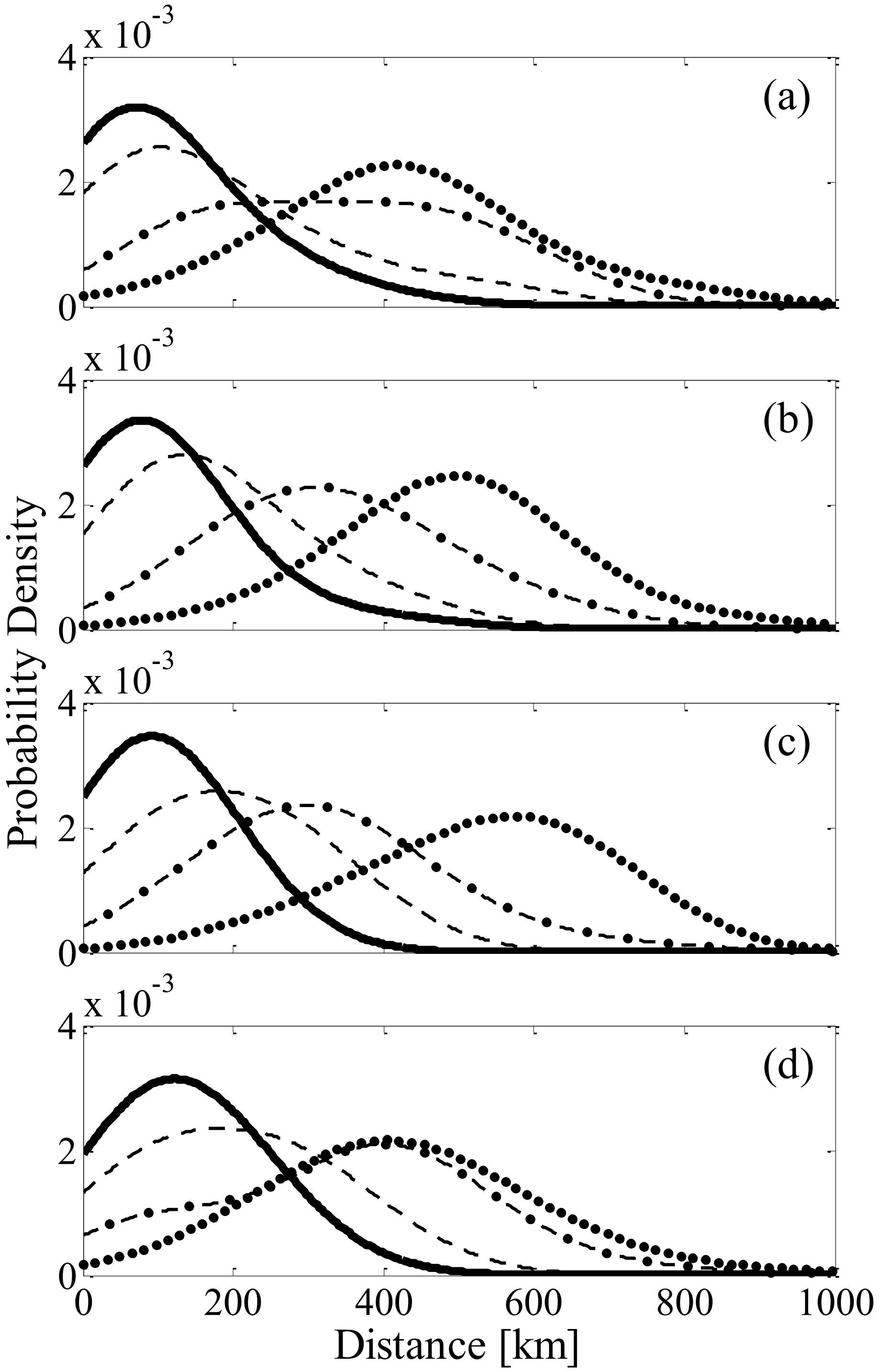

Figure S10: The dispersal kernels for particles from a) Solomon Islands, b) Vanuatu and c) New Caledonia driven by ocean currents in the depth range 800-1400m. Solid, dashed, short-dashed and dotted lines show dispersal kernels after 50, 100, 250 and 500 days respectively. Curves obtained from a normal kernel smoothing density estimate with 10,000 particles and a bandwidth of 100km.

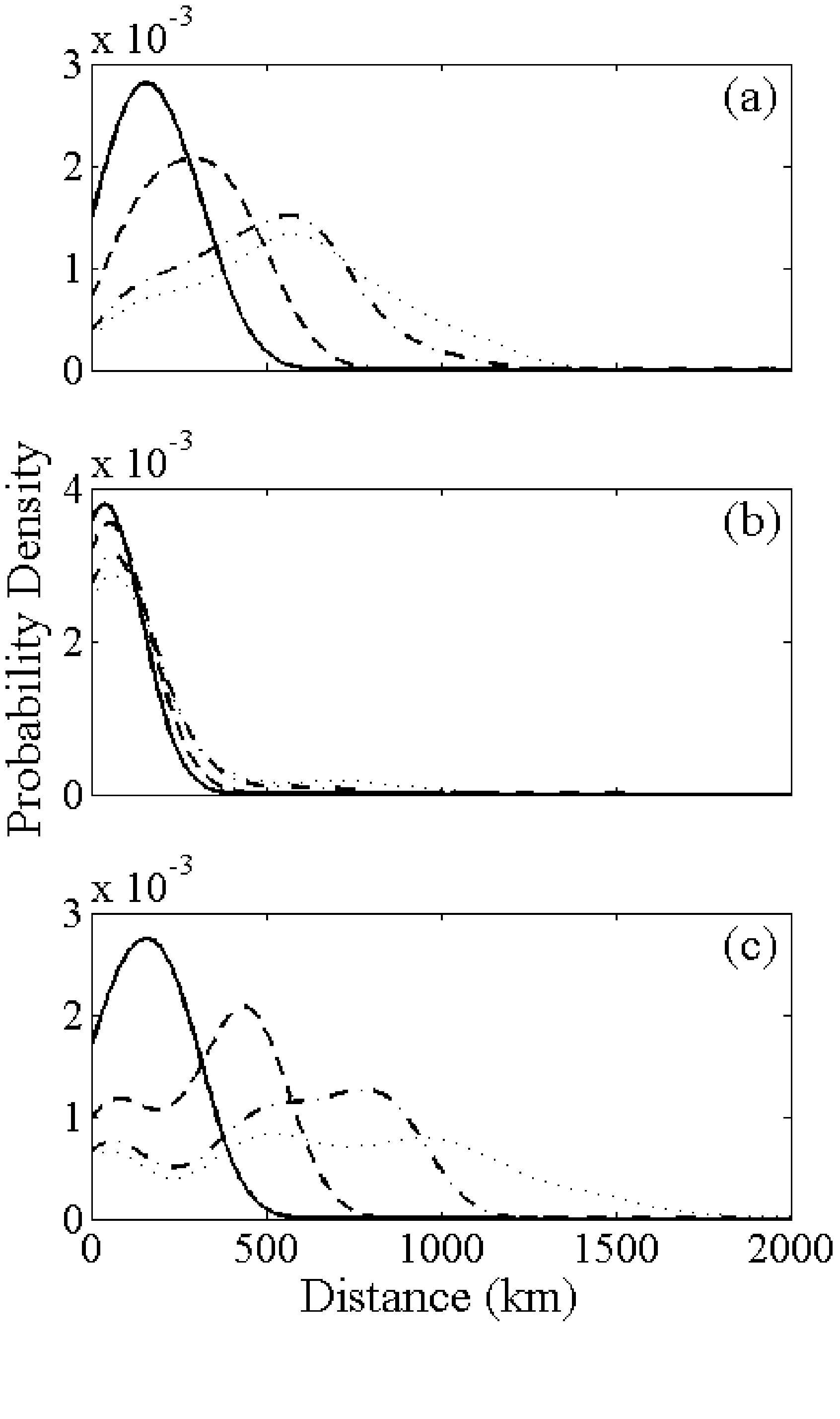

Figure S11: The dispersal kernels for particles from a) Solomon Islands, b) Vanuatu and c) New Caledonia driven by ocean currents in the depth range 1400-2500m. Solid, dashed, short-dashed and dotted lines show dispersal kernels after 50, 100, 250 and 500 days respectively. Curves obtained from a normal kernel smoothing density estimate with 10,000 particles and a bandwidth of 100km.

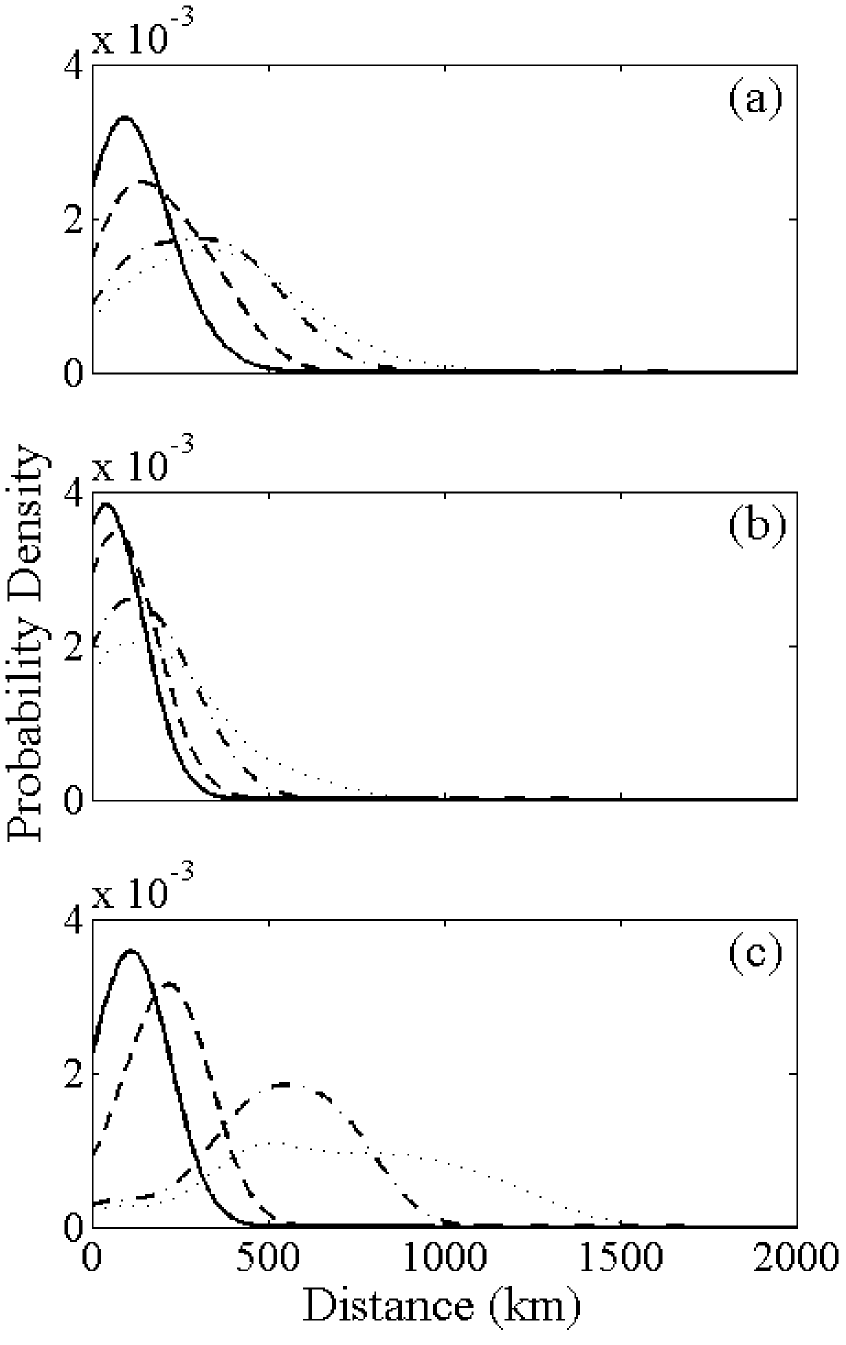

Fig S12: Particle tracks of 30,000 simulated larvae (10,000 each from Solomon Islands, Vanuatu and New Caledonia; red, green and blue tracks respectively) driven by ocean currents in the depth range 1400 - 2500m. A cyan dot marks the starting location of each particle. The tracks for 50, 250 and 500 days are shown in progressively lighter colours.

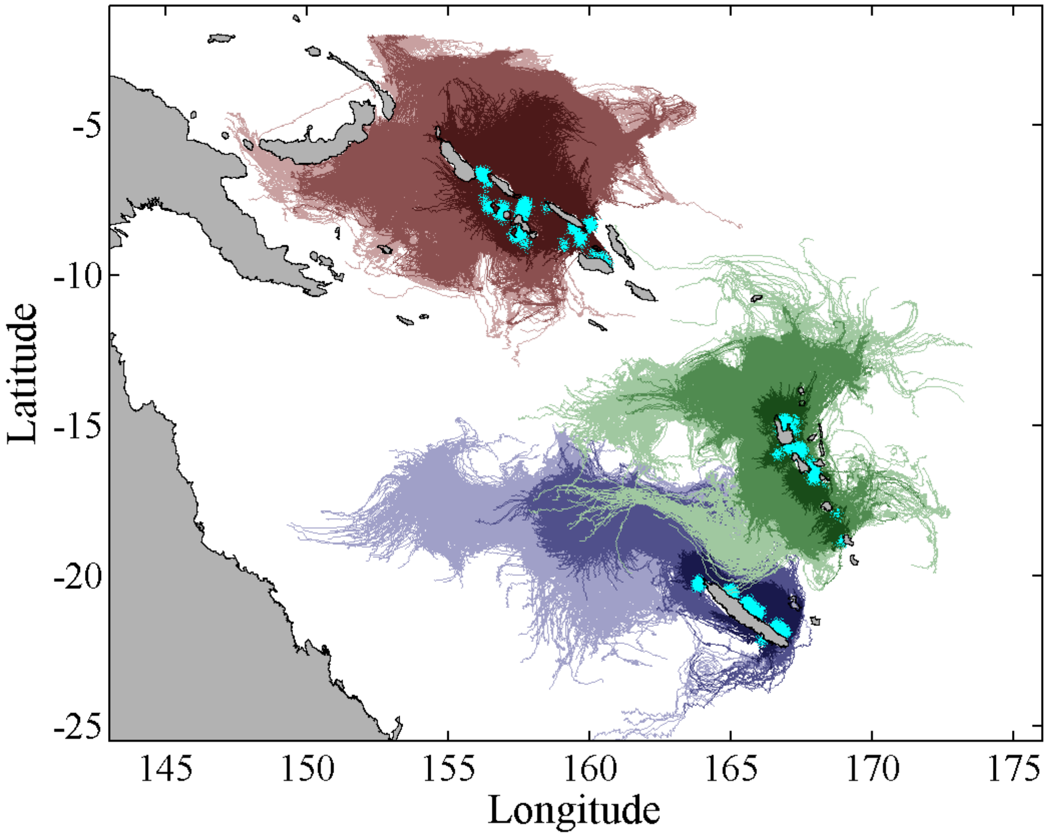

Supplement: Supporting information S1 — Interrogation of the ARGO probe data [PDF]. Results of analyses to demonstrate limited variability over seasons, depth partitions, and multi-year periods in the nine year dataset of oceanographic current data. (PDF) [file pone.0023063.s001.pdf]
