# Supplementary figures and images for: Larval Transport Modeling of Deep-Sea Invertebrates Can Aid the Search for Undiscovered Populations
Source: PLoS One. 2011 Aug 8;6(8):e23063. doi: 10.1371/journal.pone.0023063 (PMC3152551; doi:10.1371/journal.pone.0023063)

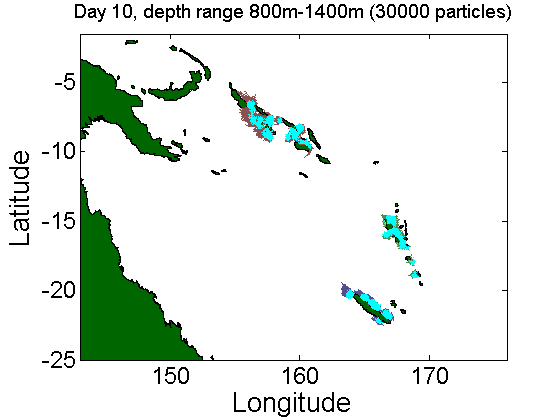

Supplement: Supporting information S2 — Dispersal pathways [animated GIF]. Animation demonstrating particle (predicted larval) pathways originating at the source populations of the model organism, chitons. (GIF) [file pone.0023063.s002.gif]
